# The Inflammatory Microenvironment in Hepatocellular Carcinoma: A Pivotal Role for Tumor-Associated Macrophages

**DOI:** 10.1155/2013/187204

**Published:** 2012-12-30

**Authors:** Daria Capece, Mariafausta Fischietti, Daniela Verzella, Agata Gaggiano, Germana Cicciarelli, Alessandra Tessitore, Francesca Zazzeroni, Edoardo Alesse

**Affiliations:** Department of Biotechnological and Applied Clinical Sciences, University of L'Aquila, Via Vetoio, Coppito II, 67100 L'Aquila, Italy

## Abstract

Hepatocellular carcinoma (HCC) is one of the most common and aggressive human cancers worldwide. HCC is an example of inflammation-related cancer and represents a paradigm of the relation occurring between tumor microenvironment and tumor development. Tumor-associated macrophages (TAMs) are a major component of leukocyte infiltrate of tumors and play a pivotal role in tumor progression of inflammation-related cancer, including HCC. Several studies indicate that, in the tumor microenvironment, TAMs acquire an M2-polarized phenotype and promote angiogenesis, metastasis, and suppression of adaptive immunity through the expression of cytokines, chemokines, growth factors, and matrix metalloproteases. Indeed, an established M2 macrophage population has been associated with poor prognosis in HCC. The molecular links that connect cancer cells and TAMs are not completely known, but recent studies have demonstrated that NF-*κ*B, STAT-3, and HIF-1 signaling pathways play key roles in this crosstalk. In this paper, we discuss the current knowledge about the role of TAMs in HCC development, highlighting the role of TAM-derived cytokines, chemokines, and growth factors in the initiation and progression of liver cancer and outlining the signaling pathways involved in the interplay between cancer cells and TAMs.

## 1. Introduction

Hepatocellular carcinoma (HCC) is one of the most aggressive human cancers and the third leading cause of death worldwide [1]. Despite the recent advance in diagnosis and treatment of HCC, it remains a highly lethal disease due to recurrence of metastasis [2]. 

HCC is an example of inflammation-related cancer, as the chronic inflammatory state appears to be necessary for the initiation and development of liver cancer. Several studies have shown that chronic infections with hepatitis viruses (hepatitis B virus, HBV and hepatitis C virus, HCV) are major risk factors for HCC development. Other risk factors to liver carcinogenesis include chronic alcohol abuse, biliary disease, metabolic disorders, drugs, toxins, and genetic conditions, such as hereditary hemochromatosis and 1-antitrypsin deficiency [3]. 

The chronic inflammation is characterized by the continued expression of cytokines and recruitment of immune cells to the liver. Activated inflammatory cells release free radicals, such as reactive oxygen species (ROS) and nitric oxide (NO) reactive species, which in turn can cause DNA damage and lead to gene mutations, thus fostering neoplastic transformation. In fact, hepatic oxidative stress is also strongly associated with increased risk for HCC in patients with chronic HBV and HCV infections [3, 4]. The chronic inflammation also affects many cellular pathways, leading to fibrosis and cirrhosis and finally hepatocarcinogenesis. Liver injury induces tissue repair and liver regeneration, which involve deregulated growth and death of hepatocytes. High cell turnover induces several critical alterations for malignant transformation, including structural and/or functional modifications of proteins involved in cell-cycle control, apoptosis, oxidative stress, lipid peroxidation and DNA repair damage [5, 6]. Moreover, Tumor-Necrosis-Factor-alpha (TNF-*α*)-induced Nuclear Factor kappa B (NF-*κ*B) activation plays a key role in hepatocarcinogenesis [7, 8].

**Figure 1:**
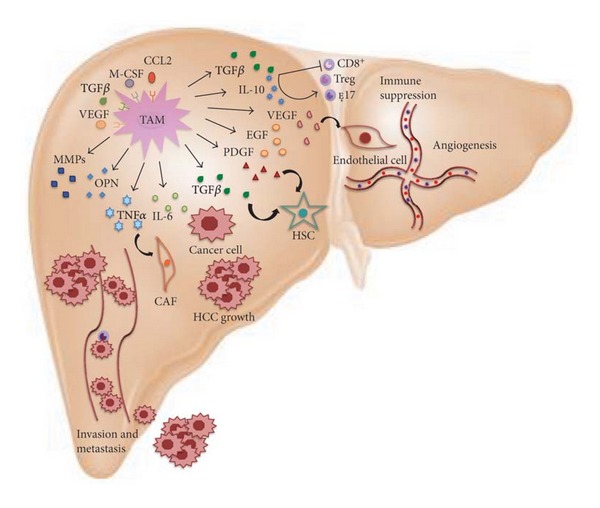
The roles of tumor-associated macrophages (TAMs) in liver cancer. TAMs promote hepatocellular carcinoma (HCC) growth, angiogenesis, invasion, and metastasis, as well as the suppression of antitumor immune response by interacting with both stromal and cancer cells within the tumor microenvironment. TAMs are recruited in HCC milieu by M-CSF, CCL2, VEGF, and TGF*β*, and they, in turn, release many cytokines, chemokines and growth factors, which are implicated in such crosstalk. In particular, IL-6 and TGF*β* favor tumor growth, whereas TNF*α*, OPN, MMPs, and IL-6 are involved in invasion and metastasis; TGF*β*, in concert with IL-10, promotes the suppression of antitumor immune response. Finally, angiogenesis is induced by several molecules, including VEGF, EGF, PDGF, and TGF*β*. Refer to the text for abbreviations.

It is therefore clear that the crosstalk between tumor cells and their surrounding microenvironment is required for HCC development. The tumor microenvironment plays critical roles in modulating the process of liver fibrosis, hepatocarcinogenesis, epithelial-mesenchymal transition (EMT), tumor invasion, and metastasis. HCC microenvironment consists of (a) stromal cells, such as carcinoma-associated fibroblasts (CAFs), hepatic stellate cells (HSCs), endothelial cells and immune cells, (b) growth factors and inflammatory cytokines, and (c) extracellular matrix proteins [1]. Tumor-associated macrophages (TAMs) are a major component of leukocyte infiltrate and play a crucial role in this scenario, by producing signal molecules, which promote and sustain tumor progression (Figure 1). This review focuses on the activities that TAMs exert in HCC, paying close attention to cytokines, chemokines, and growth factors secreted by TAMs, as well as to intracellular signaling pathways that play pivotal roles in regulating TAM functions and TAM-cancer cell crosstalk. A better understanding of the molecular events underlining the relationship between cancer cells and TAMs may be useful for the discovery of novel therapeutic targets.

## 2. TAMs and HCC

**Figure 2:**
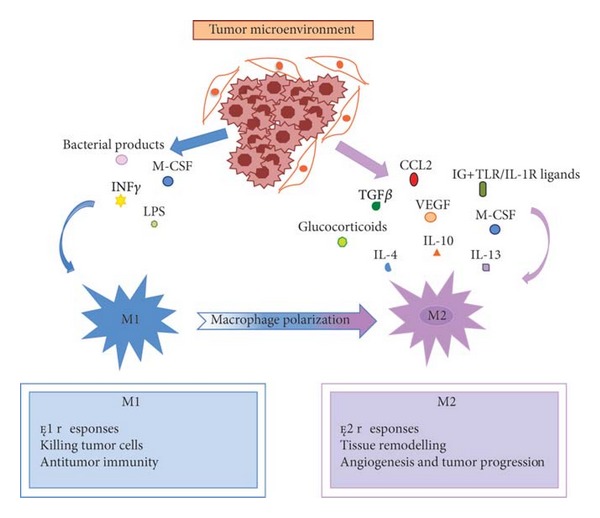
The phenotypic polarization of macrophages in the tumor microenvironment. Macrophages can be schematically classified into two main classes depending on their phenotypic polarization: macrophages mount M1 phenotype in response to M-CSF, INF*γ*, LPS and other microbial products, whereas they differentiate into M2 in the presence of TGF*β*, VEGF, CCL2, M-CSF, IL-4, IL-10, IL-13, glucocorticoids and immune complexes/TLR ligands. M1 and M2 display different functions. M1 macrophages are able to trigger Th1 immune response and exert cytotoxic activity towards ingested microorganisms and cancer cells. M2 macrophages activate Th2 immune response and promote angiogenesis, tissue remodeling, and tumor progression. Refer to the text for abbreviations.

Among the immune cell types present within the HCC, TAMs play a leading role in the setting of the crosstalk between tumor and stromal cells [9]. Indeed, TAMs are key actors of cancer-related inflammation, representing the main type of inflammatory cells infiltrating tumors [10]. TAMs originate from circulating monocytic precursors, which are recruited within the tumor microenvironment by tumor-derived signals, including Chemokine (C-C motif) Ligand 2 (CCL2) and Macrophage Colony-Stimulating Factor (M-CSF), and differentiate into mature macrophages. Thanks to their plasticity, macrophages are able to exert both anti- and protumor activities through the expression of different functional programs in response to distinct microenvironmental signals [11]. Accordingly with the phenotypic polarization, macrophage state of activation can be classified as M1 (or classical activated) or M2 (or alternatively activated) (Figure 2) [12, 13]. Macrophages mount M1 phenotype in response to microbial stimuli and Interferon gamma (INF-*γ*): classical activation of macrophages is characterized by high capability to present antigen and high expression of Interleukin 12 (IL-12) and other proinflammatory cytokines, thus being able to trigger T helper 1 (Th1) immune response [14, 15]. They have cytotoxic activity towards ingested microorganisms and cancer cells, by producing high amounts of toxic intermediates, such as NO and ROS [14, 15]. On the other hand, when monocytes are exposed to Interleukin 4 (IL-4), Interleukin 13 (IL-13), Interleukin 10 (IL-10), glucocorticoids, and immune complexes/Toll-like Receptor (TLR) ligands, they polarize towards M2 phenotype, characterized by poor antigen presenting capability, expression of a distinctive set of cytokines and chemokines, such as IL-10, Transforming Growth Factor *β* (TGF-*β*) Chemokine (C-C motif) Ligand 17 (CCL17), Chemokine (C-C motif) Ligand 22 (CCL22), and Chemokine (C-C motif) Ligand 24 (CCL24). M2 macrophages activate T helper 2 (Th2) immune response and promote angiogenesis, tissue remodeling and repair [10]. Further, M2 macrophages express specific changes in some metabolic pathways: arginine metabolism is orientated toward the production of ornithine and polyamine by arginase I and II instead of citrulline and NO by inducible nitric oxide synthase (iNOS) [16, 17]. However, it is clear from previous studies that the different functional patterns mounted by macrophages in response to distinct microenvironmental signals do not display a precise dichotomy between M1 and M2 responses [15, 18]. In this regard, Stout et al. proposed a model of functional adaptivity, suggesting not only that macrophages can adapt to microenvironmental signals by mounting different functional pattern, but also that they can progressively change their functional phenotype in response to progressive variation of these signals [18].

Within the tumor microenvironment, TAMs are mainly polarized towards M2 phenotype. In fact, TAMs were reported to express high levels of IL-10 and arginase I and low levels of proinflammatory cytokines, NO and ROS. Furthermore, TAMs are poor antigen-presenting cells [10, 13]. Notably, arginase expression by TAMs was previously suggested to be an adaptor mechanism to avoid formation of cytotoxic NO concentrations in tumors [19]. Mouse macrophages overexpressing arginase promoted proliferation of tumor cells *in vivo* [20] and macrophage-dependent tumor vascularization required polyamine synthesis [21].

Several clinical studies have shown that increased number of TAMs frequently correlate with angiogenesis, metastasis, and poor prognosis [14, 22, 23]. Nevertheless, there is a consensus view that macrophage polarization is strongly related to tumor stage, suggesting that a dynamic switching from M1 phenotype during the early phases of chronic inflammation to M2-like one in established tumors might occur. Moreover, several studies observed a “mixed” phenotype-expressing TAM population in different established murine and human tumors [24–29]. For instance, Sugai et al. observed increased levels of both IL-12 and IL-10 in monocytes from progressed gastric cancer patients with respect to healthy donors [27]. The hallmark of plasticity is also highlighted by the fact that the phenotype of macrophages also differs from tumor to tumor or within different areas of the same tumor. Soluble mediators secreted by cancer cells can alter the physiological development of macrophages, triggering transient early activation of monocytes in peritumoral stroma, and inducing immunosuppressive macrophages in cancer nests [30]. According to this model, tumor cells reeducate macrophages to adopt specific phenotype depending on the tumor area in which they localize: monocytes in peritumoral stroma are rapidly activated after their first exposure to tumor microenvironment, while the same cells become exhausted when they are in close proximity of tumor cells, thus failing to trigger an effective antitumor immune response [31]. In human HCC, most of the macrophages localized in the peritumoral stromal region mount an activated phenotype, with high expression of HLA-DR (Human Leukocyte Antigen), Interleukin 1 beta (IL-1*β*), Interleukin 6 (IL-6), and Interleukin 23 (IL-23), whereas they exhibit a HLA-DR^low^IL-10^low^ phenotype in the cancer nests [30, 32].

The importance of analyzing macrophage activation state is due to the fact that the type of macrophage polarization at the tumor site represents a prognostic factor. In HCC, tumor cells have been demonstrated to recruit and activate TAMs by secretion of Vascular Endothelial Growth Factor (VEGF), Platelet-derived Growth Factor (PDGF), TGF-*β*, CCL2, or M-CSF [33–35]. The expression of glypican-3 on the surface of liver cancer cells seems to be also implicated in TAM recruitment [36]. Recent studies have reported high expression of M-CSF in peritumoral liver tissue, which was associated with macrophage density, intrahepatic metastasis, and poor survival after hepatectomy [34, 37]. Moreover, a higher mRNA level of CCL2 is found in human HCC and the inhibition of Akt/NF-*κ*B-induced CCL2 production resulted in a reduced migration and invasion of HCC cell lines [38, 39].

The upregulation of M2-associated genes in HCC-infiltrating macrophages, such as CD163, Fc fragment of IgG, and C-type lectin domain, has been recently demonstrated by examining gene expression profile [40]. Moreover, an established M2 macrophage population has been associated with poor prognosis in HCC [41]. The association between TAM density and unfortunate prognosis has been confirmed by several works [34, 42–44]. However, Li et al. observed that increased TAM infiltration was associated with improved overall survival after tumor resection, suggesting that TAMs could protect HCC patients from recurrence and metastasis [45]. Similarly, Chew et al. have shown that high levels of proinflammatory molecules derived from tumor-infiltrating cells were associated with a better survival in HCC patients [46]. Despite this latter experimental evidence, the recruitment and activation of TAMs are considered key events in HCC progression due to their secretion of soluble mediators, which in turn sustain tumor cell survival, proliferation and dissemination. In fact, Kuang et al. demonstrated that the inhibition of monocytes/macrophages inflammation in hepatoma-bearing mice through macrophage depletion experiments markedly reduced tumor growth [32]. Moreover, macrophages play also a role in the implantation of metastatic niches at distant sites of primary tumor; these niches seems to be a reservoir of myeloid derived cells, where monocytes would be rapidly mobilized to differentiate into macrophages in response to tumor signals, thus amplifying metastatic seeding and growth [13]. In this regard, Wyckoff et al. demonstrated that genetic reduction of TAMs resulted in a decrease of circulating tumor cells in a mouse model of breast cancer [47].

## 3. TAM-Derived Factors in HCC

### 3.1. TAM-Derived Cytokines and Chemokines

Inflammatory milieu has been established as a crucial component of HCC development and the crosstalk between tumor and stromal cells plays crucial roles in the regulation of HCC progression [1]. TAMs are involved in a complex interrelationship with cancer cells, and TAM-released cytokines and chemokines play an important role in the initiation and progression of liver cancer, regulating tumor growth, invasion, and metastasis. Inflammatory cytokines profile has been recognized to have a prognostic value. In fact, the expression of inflammation-associated genes, such as IL-6 and TNF-*α*, in peritumoral liver tissue was reported to predict late HCC recurrence [48, 49].

#### 3.1.1. IL-6

IL-6 is a pleiotropic cytokine with a wide range of biological functions in immune regulation, inflammation, and oncogenesis. IL-6 plays a crucial role in the pathogenesis of HCC. In a chemically-induced HCC mouse model, diethylnitrosamine (DEN) exposure promotes IL-6 production by Kupffer cells in response to Interleukin 1 alpha (IL-1*α*) release from damaged hepatocytes). In turn, IL-6 promotes abnormal compensatory proliferation of surviving hepatocytes, thus stimulating the initiation and development of HCC. IL-6 exerts its oncogenic activity by triggering downstream Signal Transducer and Activator of Transcription 3 (STAT-3) and Extracellular-signal-Regulated Kinases (ERK) pathways, which in turn control target genes involved in both cell proliferation and survival [50]. In this model, IL-6^−/−^ mice developed fewer tumors than wild type controls [51]. Estrogens suppress IL-6 production by Kupffer cells, partially explaining the gender bias in liver cancer [51]. Moreover, high IL-6 expression and the activation of IL-6 signaling pathway lead to HCC development in obese mice, representing a link between obesity and HCC [52]. Increased serum levels of IL-6 have been associated with high risk to develop HCC in patients with chronic hepatitis B and C; accordingly, high serum IL-6 has been frequently observed in patients with HCC and it was associated with a poor prognosis [53–55]. Furthermore, IL-6 showed prometastatic properties, and an early study demonstrated that exogenously administered IL-6 affected the metastatic potential of rat hepatocellular carcinoma cells [56]. Increased IL-6 serum levels could also be used to differentiate primary or metastatic HCC from benign lesions [57]. TAM-derived IL-6 seems also to favor the epithelial-mesenchymal transition of HCC, which has an important role in tumor progression [58, 59]. Indeed, IL-6 is believed to have immunosuppressive properties and to affect, in concert with other cytokines, T cell subset differentiation [60]. Kuang et al. showed that TAM-released proinflammatory cytokines IL-6 and IL-1*β* promoted T helper 17 (Th17) cell expansion in HCC, whereas Zhang et al. reported that high levels of Th17 cells correlated with microvessel density and poor survival in HCC patients [32, 61]. 

#### 3.1.2. TNF-*α*

TNF-*α* is mainly produced by macrophages and is fundamental for liver regeneration following liver injury or partial hepatectomy [62]. TNF-*α* is strongly involved in the pathogenesis of HCC, promoting invasion, angiogenesis, and metastasis [23]. In fact, TNF-*α* has been shown to promote HCC in a genetic model of inflammation-induced carcinogenesis, in which mice lacking the P-glycoprotein Mdr2 developed cholestatic inflammation followed by HCC. In this model, *in vivo* administration of TNF-*α*-neutralizing antibody impaired HCC development [7]. Moreover, TNF-*α* plays a crucial role in DEN-induced HCC mouse model. TNF-*α*, in concert with IL-6, promoted hepatosteatosis and steatohepatitis, thus favoring obesity-enhanced HCC. In fact, the ablation of TNF-*α* signaling abolished HCC development in these mice [52]. TNF-*α* is associated with cell-cycle progression, tumor growth, and oxidative stress, stimulating the expression of Transforming Growth Factor alpha (TGF-*α*) in mouse hepatocytes and the formation of 8-oxodeoxyguanosine, a critical biomarker of oxidative stress and carcinogenesis [63–65]. Furthermore, TNF-*α* significantly induced phosphorylation of p38 Mitogen-Activated Protein Kinase (MAPK), ERK, Akt, and production of Interleukin 8 (IL-8) from HCC cells [66]. TAM-derived TNF-*α* also stimulated the activation of CAF, which are dominant elements in tumor inflammatory milieu, and was involved in promoting Th17 cell expansion [32, 67]. Furthermore, TNF-*α* and IL-1*β* supported tumor immune escape *in vitro*, by inducing the expression of TNF-related Apoptosis-inducing Ligand (TRAIL) on the surface of liver cancer cells, which in turn promoted apoptosis of activated T cells [68]. TNF-*α* also stimulated the expression of the negative costimulatory molecule B7 homolog 1 (B7-H1) (or Programmed Cell Death 1 Ligand 1, PDL-1) on macrophage surface, thus suppressing CD8^+^ T-cell antitumor immune response [43, 69]. The downstream principal mediator of protumoral activity of TNF-*α* is NF-*κ*B, whose target genes are involved in cell proliferation and survival [3]. Of note, TNF-*α* is also induced by NF-*κ*B in a positive feedback loop. TNF-*α* levels in HCC patients have also been investigated, but controversial data have been reported, whereas TNF-*α* (308) single nucleotide polymorphism was associated with cancer susceptibility [70].

#### 3.1.3. IL-10

IL-10 is one of the most important immunosuppressive cytokines. Several studies have reported high IL-10 levels in HCC patients, as reviewed by Budhu and Xin [5]. IL-10, along with TNF-*α*, autocrinally stimulated the expression of B7-H1 on macrophage surface, impairing CD8^+^ T cell activity and supporting tumor immune escape [43, 69]. Moreover, B7-H1 expression by liver cancer cells correlated with TAM infiltration in HCC tissue and was dependent on IL-10-induced NF-*κ*B an STAT-3 signaling pathways [71]. IL-10 is also involved in the induction of FOXP3^+^ (Forkhead Box P3) regulatory T cell (Treg) differentiation. Tregs strongly suppressed the activity of effector T cells and are associated with HCC aggressive clinicopathological features and poor survival [42, 72–74]. Moreover, high prevalence of Treg cells along with high levels of IL-10 has been observed in HCC patients [75]. In addition to play an immunosuppressive role, an increased expression of IL-10 is also correlated with high angiogenic activity in a HCC mouse model [76].

#### 3.1.4. IL-17

The proinflammatory cytokine Interleukin 17 (IL-17) has been demonstrated to foster tumor immune escape in HCC [77]. IL-17 stimulated macrophages to express inflammatory cytokines, such as IL-1*β*, IL-10 and TNF-*α*, that autocrinally upregulated B7-H1 expression; thereafter, B7-H1-bearing macrophages in the peritumoral stroma suppressed cytotoxic T cell response via B7-H1 signaling [77]. Several reports demonstrated an association between high infiltration of IL-17-producing cells in the peritumoral stroma and the progression of HCC. Therefore, IL-17-mediated immunosuppression might represent a further mechanism by which this cytokine exerts its protumoral activity in liver cancer [32, 78].

#### 3.1.5. Chemokines

In HCC progression, chemokines and their receptors play an intricate role. Chemokines could affect the recruitment of immunosuppressive Th17 cells, as reported by Kuang et al., which detected an increase of Th17 cell population in response to high local levels of CCL22 and Chemokine (C-C motif) Ligand 20 (CCL20) [78]. Furthermore, Zhang et al. observed higher levels of CCL20 in tumor tissue compared with nontumor one in HCC patients [61]. Uchida et al. reported that the incidence of intrahepatic metastasis was higher in patients with increased expression of the CCL20 receptor (CC-chemokine receptor 6, CCR6) than in patients with low expression of this molecule. The authors suggested that CCR6 might be associated with the intrahepatic metastasis of HCC and may be used as a prognostic factor after hepatic resection for HCC [79]. In addition, Shih et al. identified CCR6 as a marker of endothelial progenitor cells in tumor [80]. Indeed, M2-like TAMs produce several chemokines such as CCL17, CCL22, and CCL24, that interact with their receptors expressed mainly by Th2 and Treg cells, promoting the recruitment of these ineffective T cell subsets [16]. The Chemokine (C-X-C motif) Ligand 12 (CXCL12) is involved in Treg recruitment. In fact, Shen et al. reported that CXCL12 was responsible for the increased recruitment of Treg cells to tumor sites in HCC patients by the activation of CXCL12/CXC-Chemokine Receptor 4 (CXCR4) signaling [75]. The CXCL12-CXCR4 axis has been demonstrated to promote growth, invasion and metastasis of HCC cell lines [81, 82]. CXCL12-CXCR4 system is also involved in the secretion of Matrix Metalloprotease 9 (MMP-9) and Matrix Metalloprotease 2 (MMP-2), thus favoring metastasis [83]. Schimanski et al. also reported that strong expression of CXCR4 was significantly associated with progressed HCC, correlating with distant dissemination of lymphatic metastasis and a reduced 3-year-survival rate [84]. Recently, up-regulation of IL-8 receptor CXC-Chemokine Receptor 2 (CXCR2) was found in HCC patients and correlated with intrahepatic metastasis [85].

### 3.2. TAM-Derived Growth Factors

The deregulated expression of growth factors and the activation of their signaling pathways are hallmarks of chronic inflammatory liver diseases and HCC [3]. Aberrant growth factors expression contribute to neoplastic transformation and to the maintenance of acquired tumoral phenotype of HCC cells [86]. Moreover, TAM-derived growth factors strongly affect angiogenesis, as confirmed by Peng et al., which showed that TAM count was correlated to microvessels density [87]. Furthermore, marginal macrophage density is associated with angiogenesis and poor prognosis [44]. In addition to hepatocytes, extracellular matrix-producing cells and inflammatory cells such as macrophages have been demonstrated to produce TGF-*β*1, PDGF, VEGF, and the Epidermal Growth Factor Receptor (EGFR) ligands (TGF-*α*, Epidermal Growth Factor (EGF) and Amphiregulin) [1, 6, 88].

#### 3.2.1. TGF-*β*

TGF-*β* exerts a dual role in liver hepatocarcinogenesis, showing both antitumoral and protumoral activities depending on HCC stage. In precancerous state, TGF-*β*1 acts as tumor suppressor, mediating anti-proliferative and proapoptotic signals, whereas in the established tumors TGF-*β*1 promotes tumorigenesis through several mechanisms [89]. First, TGF-*β*1, like IL-10, is an immunosuppressive molecule. TGF-*β*1 has been demonstrated to suppress INF-*γ* production by CD8^+^ T cells and to promote, in concert with IL-6, IL-1*β*, and IL-10, Treg generation, and Th17 differentiation, thus favoring tumor growth and progression [75, 90–92]. Moreover, TGF-*β*1 is one of the soluble factors able to activate HSCs, another crucial population within HCC microenvironment [1]. TGF-*β*1 expression is also associated with migration, invasion, angiogenesis, and metastasis. In fact, TGF-*β* induces *α*3*β*1 integrin, which is a marker of invasiveness [93, 94], as well as the secretion of VEGF [95]. In a recent preclinical study, targeting TGF-*β* has been demonstrated to affect several kinases involved in HCC cell migration control, such as SMAD-2 and focal adhesion kinase (FAK). Blocking TGF-*β* resulted in an overall inhibition of tumor growth and metastasis [96]. In HCC, TGF-*β*1 also promotes EMT through the downregulation of E-cadherin, a major component of epithelial adherent junctions, and the upregulation of the E-cadherin repressor snail and PDGF intracellular signaling [97, 98]. In accordance to the pivotal role of TGF-*β*1 in EMT, a recent work demonstrated that coupled with E-cadherin downregulation, TGF-*β*1 can induce N-cadherin, vimentin, and the HCC-associated antigen CD147 [99]. Experimental evidence in the literature reported that TGF-*β* also regulates several microRNAs involved in HCC pathogenesis, such as miR-23a, 27a, 24 and 181b [100, 101]. In particular, miR-181b has been demonstrated to up-regulate MMP-2 and MMP-9, favoring invasion and metastasis [100]. Early studies reported an increase of TGF-*β* levels in both plasma and tissue of HCC patients [102, 103]. Furthermore, TGF-*β* overexpression was associated with short survival in HCC patients [104]. Nevertheless, a recent study by Mamiya et al. reported that a decreased expression of TGF-*β* receptor type II was associated with intrahepatic metastasis, in accordance with the controversial role of TGF-*β* in HCC [105]. 

#### 3.2.2. PDGF-*β*

As mentioned above, PDGF signaling is also important in HCC. In addition to its involvement in EMT, PDGF is considered an angiogenic factor, due to its capability to stabilize blood vessels [106]. PDGF also promotes the activation of CAFs and HSCs. Moreover, PDGF induces HSC differentiation into proliferating and ECM-producing myofibroblasts, which results in increased liver fibrosis and subsequent development of HCC [107]. Moreover, in this cell population, PDGF can also induce the up-regulation of amphiregulin, in turn involved in the activation of the EGFR signaling [108]. 

#### 3.2.3. EGFR Ligands

EGFR overexpression has been observed in HCC tissue and seems to be correlated to a poor patients survival. In the same way, overexpression of its ligands has been reported in HCC tumor samples [109]. The role of EGFR pathway in HCC pathogenesis was well clarified by the use of genetically modified mouse models and confirmed by the experimental evidence that EGFR inhibition prevented chemically induced HCC in rats [3, 110]. Activation of EGFR signaling is strongly associated with angiogenesis because this pathway induces VEGF production [111]. 

#### 3.2.4. VEGF

VEGF is a critical player in liver cancer angiogenesis. VEGF exerts its effect on the proliferation of both endothelial and VEGF-A receptor-expressing cancer cells [112]. High levels of this growth factor and its receptors VEGFR1, VEGFR2, and VEGFR3 have been reported in HCC cell lines, tissue, and in the blood circulation of HCC patients. Activation of VEGF signaling has been associated with vascular invasion, HCC grade, and poor outcome and survival (as reviewed in [113]). 

### 3.3. Other TAM-Derived Mediators

In HCC, TAMs sustain invasion, angiogenesis, and metastasis through the expression of other several mediators different from cytokines, chemokines, or growth factors, including MMPs, osteopontin (OPN), and cyclooxygenase 2 (COX-2) [114]. 

With regard to MMPs, previous studies have reported an elevated expression of MMP-9 in HCC that was associated with growth and invasiveness [115–118]. A recent study by Roderferl et al. reported that MMP-9-expressing macrophages were involved in matrix remodeling and degradation at the invasive front of murine HCC [119]. Thanks to their proteolytic activity, MMP-9 and MMP-2 promote ECM-stored growth factors mobilization, including VEGF, thus favoring angiogenesis in HCC [120, 121]. Moreover, MMPs activated TGF-*β* during the EMT in HCC [122]. MMP-9 has also been demonstrated to induce HCC invasion and metastasis by cleaving and consequently activating the OPN precursor [123].

OPN is a phosphorylated acidic glycoprotein which was found to be expressed in macrophages after liver injury, taking part in the host response [124]. OPN is involved in the control of inflammation and tumor progression and contributes to HCC invasion and metastasis interacting with integrins [125]. OPN plasma levels were found increased in HCC patients and were associated with reduced liver function and worse prognosis [126]. Neutralizing OPN by anti-OPN antibodies resulted in strong inhibition of invasion and metastasis of HCC cells *in vitro *and* in vivo *[127].

COX-2 is involved in the synthesis of lipid inflammatory mediators from arachidonic acid and its overexpression has been observed in HCC patients [128, 129]. Furthermore, Cervello et al. reported that in primary HCC COX-2 expression was significantly correlated to the presence of inflammatory cells, including macrophages. The authors suggested that COX-2 expressing inflammatory cells were involved in the early stages of hepatocarcinogenesis [129]. 

In addition, TAM-derived urokinase-type plasminogen activator has been correlated with invasion and metastasis of HCC [130].

## 4. Signaling Pathways Linking TAMs and HCC

**Figure 3:**
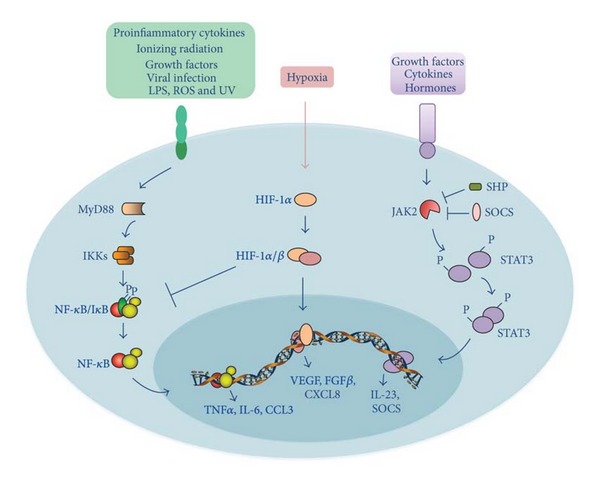
Schematic representation of NF-*κ*B, HIF-1, and STAT-3 signaling pathways linking inflammation and liver cancer. Proinflammatory signals bind to their corresponding receptors, leading to the recruitment of receptor-associated proteins, such as MyD-88. In turn, these associated proteins trigger a phosphorylation cascade that leads to activation of the IKK complex, which is responsible for the phosphorylation of the I*κ*B protein. Phosphorylated I*κ*B is degraded by proteasome, thereby allowing free NF-*κ*B dimers to translocate to the nucleus and transactivate target genes, such as TNF*α*, IL-6, and CCL3. HIF-1*α* is the central regulator of the hypoxic response. HIF-1*α* is activated by hypoxia and its activity progressively increases with a decrease in O_2_ gradient. Heterodimerization of HIF-1*α* with HIF-1*β* allows for DNA binding to hypoxia response elements (HRE) and transactivation of its target genes, such as VEGF, FGF*β*, and CXCL8. Cytokine, hormone and growth factor stimulation activates JAK2, which in turn phosphorylates STAT3, allowing its dimerization, nuclear translocation, and transactivation of target genes, such as IL-23. STAT-3 signaling is turned off by protein inhibitors, such as SHP and SOCS, which are induced by STAT-3 in a negative feedback loop. Refer to the text for abbreviations.

Although many studies have shown significant alterations in the expression and activity of different cytokines and their signaling systems in liver cirrhosis and HCC, the critical components linking inflammation and liver cancer are only beginning to be unmasked. Experimental evidence gathered in genetic mouse models over the past few years identified the transcription factor NF-*κ*B, hypoxia inducible factor 1*α* (HIF-1*α*), and STAT-3 as the major molecular players linking inflammation and cancer [131] (Figure 3).

### 4.1. NF-*κ*B

The transcription factor NF-*κ*B is a key orchestrator of innate immunity and inflammation, and recent evidence suggests that it represents a molecular link between inflammation and cancer [132]. NF-*κ*B transcription factor family consists of five members: NF-*κ*B1 (p105/p50), NF-*κ*B2 (p100/p52), RelA (p65), RelB, and c-Rel, which can form homo- or heterodimers [133]. NF-*κ*B is retained in the cytoplasm of resting cells by binding with Inhibitor of NF-*κ*B (I*κ*B) proteins and can be rapidly activated upon stimulation by I*κ*B phosphorylation. I*κ*B proteins are phosphorylated by I*κ*B kinase complex (IKK), which consist of two catalytic subunits (IKK*α* and IKK*β*) and a regulatory subunit (IKK*γ* or NEMO). Recent studies have demonstrated that NF-*κ*B can be activated in two different ways, the canonical pathway and the alternative pathway. Microbial products, such as Lipopolysaccharide (LPS), and proinflammatory cytokines, such as TNF-*α* and IL-1*β*, activate the classical pathway, leading to IKK*β*-dependent phosphorylation of I*κ*B*α* and RelA-p50 complexes activation (Figure 3). The alternative pathway is activated by lymphotoxin *β* (LT*β*), CD40 ligand (CD40L), B-cell activating factor (BAFF), and RANK ligand (RANKL) and results in activation of RelB-p52 by IKK*α*-mediated phosphorylation and processing of p100 [133]. 

Genetic studies targeting NF-*κ*B activation in liver epithelial cells and in liver macrophages have demonstrated that this factor plays a key role in HCC development. Mice with hepatocyte-specific ablation of IKK*β* [50], NEMO [134], or with overexpression of an I*κ*B*α* superrepressor [7] showed increased cell death, enhanced compensatory proliferation, predisposition to malignancy, and increased tumor susceptibility. Thus, NF-*κ*B has a tumor suppressor function in liver parenchymal cells. On the contrary, NF-*κ*B activation in liver macrophages has a protumoral significance. In fact, either genetic deletion of NF-*κ*B or IL-6 [50, 51], or the inhibition of inflammatory cytokines, such as TNF-*α* [7], determined a significant reduction in HCC tumor load. Thus, detection of dying cells by Kupffer cells induces them to release more NF-*κ*B-regulated inflammatory cytokines, which are necessary for growth and survival of malignant hepatocytes. The mechanism by which necrotic hepatocytes activate NF-*κ*B in Kupffer cells was found to depend on the release of IL-1*α* by dying cells, which activates a Myeloid-Differentiation-Factor-88 (MyD-88)-dependent signaling pathway upon binding to IL-1 receptor (IL1R) on Kupffer cells [135]. 

These studies in HCC mouse models suggested that NF-*κ*B plays a proinflammatory role in macrophages during early stages of the tumor growth. The roles of NF-*κ*B in TAMs from established HCC cancers have not been yet elucidated, but recent works have dissected its function in TAMs from established tumors in a model of murine fibrosarcoma [29, 136], mouse mammary carcinoma [137], and mouse ovarian carcinoma [138]. Defective NF-*κ*B activation was identified as signature of tumor-promoting, M2-like TAMs in fibrosarcoma. In fact, several NF-*κ*B-regulated genes such as IL-12p40, TNF-*α*, Chemokine (C-C motif) Ligand 3 (CCL3), and IL-6 were downregulated in TAMs upon LPS treatment *in vitro* [29], and Saccani et al. showed that this phenotype was dependent on high levels of transcriptional inactive p50/p50 homodimers [136]. p50/p50 homodimers can compete with p50/p65 transcriptional active complex for the binding to promoter regions of proinflammatory genes. In this work, the authors showed that p50^−/−^ TAMs have a proinflammatory, anti-tumor M1 phenotype, resulting in reduced tumor growth [136]. Indeed, Colombo et al. showed that activation of NF-*κ*B is associated with mouse mammary tumor regression [137]. By contrast, a recent work by Hagemann et al. has demonstrated that inhibition of IKK*β* (and therefore of NF-*κ*B) in TAMs promotes an M1-like phenotype, whereas functional IKK*β*/NF-*κ*B activation maintains these cells in an alternative, tumor-promoting M2 phenotype [138]. These apparent discrepancy between Sica et al. studies on p50^−/−^ TAMs and Hagemann et al. work on IKK*β* ^−/−^ TAMs can be due to the different tumor types and experimental approaches utilized, as reviewed in [139]. In fact, it is well known that macrophage phenotype can differ markedly between different cancers [140]. Moreover, macrophages are very plastic cells, able to modify quickly their phenotype depending on the tumor stage [141]. 

### 4.2. STAT-3

STAT-3 is a member of the signal transducer and activator of transcription (STAT) and has been identified as one of the master regulator of macrophage transcriptional programs [142]. STAT-3 pathway is rapidly activated by several cytokines (i.e., IL-6), hormones, and growth factors (i.e., EGF and VEGF). The interaction of these ligands with their receptors triggers Janus Kinases (JAK) activation, especially JAK2, which, in turn, phosphorylates STAT-3 on the critical Tyr75 residue. The phosphorylation of STAT-3 at Tyr75 mediates STAT-3 dimerization, nucleus translocation, and DNA-binding of target genes involved in proliferation, survival, angiogenesis, and metastasis [143] (Figure 3). COX-2 has also been demonstrated to activate STAT-3 signaling pathway [144]. In normal condition STAT-3 activation is a transient and tightly regulated event. In fact, STAT-3 signaling is turned off by protein inhibitors, including SH2-containing phosphatases (SHP) and the suppressors of cytokine signaling (SOCS), which are activated by STAT-3 in a negative feedback loop [143] (Figure 3). The constitutive activation of STAT-3 signaling pathway was associated with M2 phenotype and it has been observed in both cancer and tumor-infiltrating inflammatory cells, including TAMs [3, 14, 145]. STAT-3 blocks IL-12p35 expression by dendritic cells and promotes the expression of the protumoral Interleukin 23 (IL-23) cytokine by TAMs [146]. STAT-3 activation in immune cells also exerts an immunosuppressive effect, thus counteracting anti-tumor immune response [147]. High STAT-3 levels have been detected in a large number of HCC samples and have been associated with invasiveness and poor prognosis [148–150]. Although the exact molecular events that lead to STAT-3 activation in human HCC are not well understood, NF-*κ*B-regulated IL-6 released by TAMs seems to be the major STAT-3 activator. Accordingly, IL-6^−/−^ mice showed a reduced STAT-3 activation and were less susceptible to develop DEN-induced HCC [51]. Moreover, a sixfold reduction in DEN-induced HCC load was observed in hepatocyte-specific STAT-3 deficient mice (*Stat*3^∆hep^) [151]. Furthermore, *Stat*3^∆hep^ mice developed smaller tumors than control mice, suggesting a role for STAT-3 in hepatocyte proliferation and survival [151]. The pivotal role of JAK/STAT-3 pathway in inflammation-related liver cancer was confirmed by SOCS knocking out studies [152–154]. In fact, *Socs*3^−/−^ mice were more susceptible to DEN-induced HCC and developed tumors increased in number and size [153, 154]. Furthermore, the inhibition of STAT-3 and NF-*κ*B signaling pathways blocks the TAM-induced upregulation of B7-H1 on HCC cells [71]. 

Targeting STAT-3 signaling pathway might be a hopeful approach in the treatment of HCC. Liu et al. demonstrated that blocking STAT-3 phosphorylation with specific small molecule inhibitors causes apoptosis in HCC cell lines, whereas *Stat3* antisense oligonucleotide strongly inhibited growth and metastasis of HCC *in vivo* [155, 156]. 

### 4.3. HIF-1

Hypoxic areas are often found in many solid tumors, including HCC. In addition, TAMs have been shown to accumulate in these poorly vascularized regions [157, 158]. Under low oxygen conditions, both tumor cells and macrophages mount a proangiogenic program mediated by Hypoxia Inducible Factor 1 (HIF-1). HIF-1 is a transcriptional activator complex constituted with two types of subunits, an inducible alpha subunit (HIF-1*α*, HIF-2*α*, or HIF-3*α*), and one constitutively expressed HIF-1*β* subunit. Hypoxia stabilizes HIF-1*α*, preventing its posttranslational hydroxylation and consequently proteasome-mediated degradation. In addition, hypoxia promotes HIF-1*α* association with HIF-1*β*, as well as cofactor recruitment [159] (Figure 3). HIF-1*α* is also transcriptionally regulated by NF-*κ*B, as demonstrated by Rius et al. [160]. The authors reported that bone marrow-derived macrophages upregulated NF-*κ*B, which in turn induced HIF-1*α* following short-term exposure to hypoxia. In addition, Maeda et al. observed the downregulation of HIF-1*α* in mice lacking IKK*β* in both hepatocytes and Kupffer cells [134]. TAM adaptation to hypoxia is mediated by the induction of HIF-1 and HIF-2-regulated genes, including VEGF, Fibroblast Growth Factor beta (FGF-*β*), and IL-8, as well as glycolytic enzymes [161, 162]. Furthermore, HIF-1 pathway has been demonstrated to play a role in TAM recruitment and activation. In fact, hypoxia affected the localization of both tumor and stromal cells by up-regulating their expression of CXCR4 receptor, CXCR4 ligand, and CXCL12 [163, 164]. The pivotal role of HIF-1 in sustaining oncogenic activities of TAMs has been demonstrated by HIF1*α* and HIF2*α* ablation experiments [165–167]. The lack of HIF-1*α* impaired macrophage motility and migration through extracellular matrix, as well as their suppressive abilities [165, 166]. The loss of HIF-2*α* ablation in macrophages affected TAM recruitment in a mouse model of HCC and it was associated with reduced tumor cell proliferation. In the same work, the authors demonstrated that HIF-2*α* regulated proinflammatory cytokine expression, including IL-6, by binding their promoter regions [167]. Thus, the ablation of both HIF-1*α* and HIF-2*α* resulted in reduced tumor volume and progression in different cancer models, suggesting that HIF-1*α* and HIF-2*α* regulate overlapping pathways. As suggested for NF-*κ*B and STAT-3, HIF-1 might be a potential target in HCC therapy. In fact, two different works reported that antisense HIF-1 therapy was effective in inhibiting HCC cell proliferation as well as in enhancing chemotherapy antitumor efficacy in a xenograft model of liver cancer [168, 169]. 

## 5. Conclusions and Perspectives

The crucial role of the tumor microenvironment in HCC pathogenesis is now widely accepted. TAMs represent one of the main tumor-infiltrating immune cells that sustain tumor progression. In fact, several experimental evidence showed that TAMs promote cancer cell growth, invasion, and metastasis by stimulating proliferation, survival, and EMT transition of cancer cells, as well as, by inducing angiogenesis, ECM remodeling and suppression of antitumor immunity. Thus, targeting tumor-stroma interactions is believed to be an attractive therapeutic strategy in the management of HCC. To date, most of the therapeutics have been designed to block receptors and downstream signaling pathways, so inhibiting stroma-derived protumoral signals, such as the kinase inhibitor drugs. Sorafenib, an oral inhibitor of VEGFR-2/3, PDGFR, and Raf kinases, has been demonstrated to be effective and safe in Phase III clinical trials and it is the standard therapeutic agent for advanced HCC [170, 171]. Moreover, a recent study by Zhang et al. showed that sorafenib is more effective when used in combination with zoledronic acid and clodronate-encapsulated liposomes, which deplete macrophages population [172]. A phase II clinical study of sorafenib and zoledronic acid to treat advanced HCC is currently recruiting participants. Similarly, targeting TGF-*β* signaling has been demonstrated to be a potential therapy in HCC, because TGF-*β* receptor I kinase inhibitor LY2109761 has been reported to inhibit HCC migration *in vitro* and tumor growth, intravasation, and metastasis *in vivo *[96]. Other drugs belonging to kinase inhibitors class, such as brivanib (which targets VEGFR2 and FGFR1), linifanib (which targets PDGFR and VEGFR), sunitinib (which targets PDGFR, VEGFR, c-Kit, and Flt-3), erlotinib (which targets EGFR), and PI-88 (which targets heparanase and sulfatases) are currently being investigated for efficacy and safety in liver cancer-phase III clinical trials. Similarly, efficacy and safety of recombinant monoclonal antibodies against VEGF (bevacizumab), VEGFR2 (ramucirumab), and EGFR (cetuximab) are now evaluated in phase II-III clinical trials for the treatment of HCC (as reviewed in [1]). Moreover, on the basis of experimental evidence, targeting signaling pathways involved in the crosstalk between stromal and tumor cells might also be an effective strategy against HCC. 

Thus, clarifying the molecular mechanisms underlying the crosstalk between tumor and stromal cells within HCC microenvironment will be beneficial to identify new targets for liver cancer therapy.
